# Development of a digital energy modulation framework in projectional radiography

**DOI:** 10.1002/acm2.70320

**Published:** 2025-10-24

**Authors:** Richard Ryan Wargo, William C. Sleeman, Siyong Kim

**Affiliations:** ^1^ Department of Radiology Virginia Commonwealth University Richmond Virginia USA; ^2^ Department of Radiation Oncology Virginia Commonwealth University Richmond Virginia USA; ^3^ Intelligent Health Solutions LLC Henrico Virginia USA

**Keywords:** deep learning, digital energy modulation, DRR, image translation, spectral imaging

## Abstract

**Purpose:**

Digital energy modulation is a novel framework with the potential to enhance projectional x‐ray imaging by enabling translation between different x‐ray energy domains. We evaluate the feasibility of integrating machine learning methods into this approach by leveraging digitally reconstructed radiographs (DRRs) generated from dual‐energy CT datasets.

**Methods:**

DRRs were created in 15° increments from 0° to 90°, producing 3500 images per energy domain (2 polyenergetic, 4 monoenergetic). A supervised deep‐learning approach was used to train models for energy translation, focusing on conversions between polyenergetic domains and from polyenergetic to monoenergetic images. Model performance was assessed using peak signal‐to‐noise ratio (PSNR), structural similarity index (SSIM), mean squared error (MSE), and mean absolute percentage error (MAPE). Cross‐validation and projection‐specific dataset splits were used for evaluation.

**Results:**

The models trained using cross‐validation on the various energy translations achieved the following results: PSNR: 29.1 ± 2.0, SSIM: 0.947 ± 0.017, MSE: 169.1 ± 68.3, MAPE: 8.2% ± 1.8%. When translating between polyenergetic high‐energy and low‐energy domains in projection‐specific datasets (anterior‐posterior [0°] and lateral [90°] views), models achieved the following results: PSNR: 27.4 ± 0.5, SSIM: 0.909 ± 0.003, MSE: 195.9 ± 39.7, MAPE: 10.4% ± 2.1%.

**Conclusion:**

These findings demonstrate the feasibility of a digital energy modulation framework for projectional x‐ray imaging using machine learning for energy translation. The results support the potential of this approach to enhance projectional x‐ray imaging, though future work is needed to refine the models and further explore clinical applications.

## 1 INTRODUCTION

Medical imaging relies on optimizing both image acquisition parameters and display settings to achieve high diagnostic value. In radiology and radiation oncology, basic contrast adjustments are often performed post‐acquisition using contrast and frequency processing techniques. Modifying window/level (W/L) settings is the most common adjustment, which changes how grayscale values are displayed across an entire image. However, these global adjustments can be limiting in cases where multiple tissues or specific regions of interest (ROIs) exhibit different attenuation and contrast properties.^1^, ^2^, ^3^ This often requires separate settings to optimally visualize each structure or region, which can be impractical or inefficient in clinical workflows.

**FIGURE 1:**
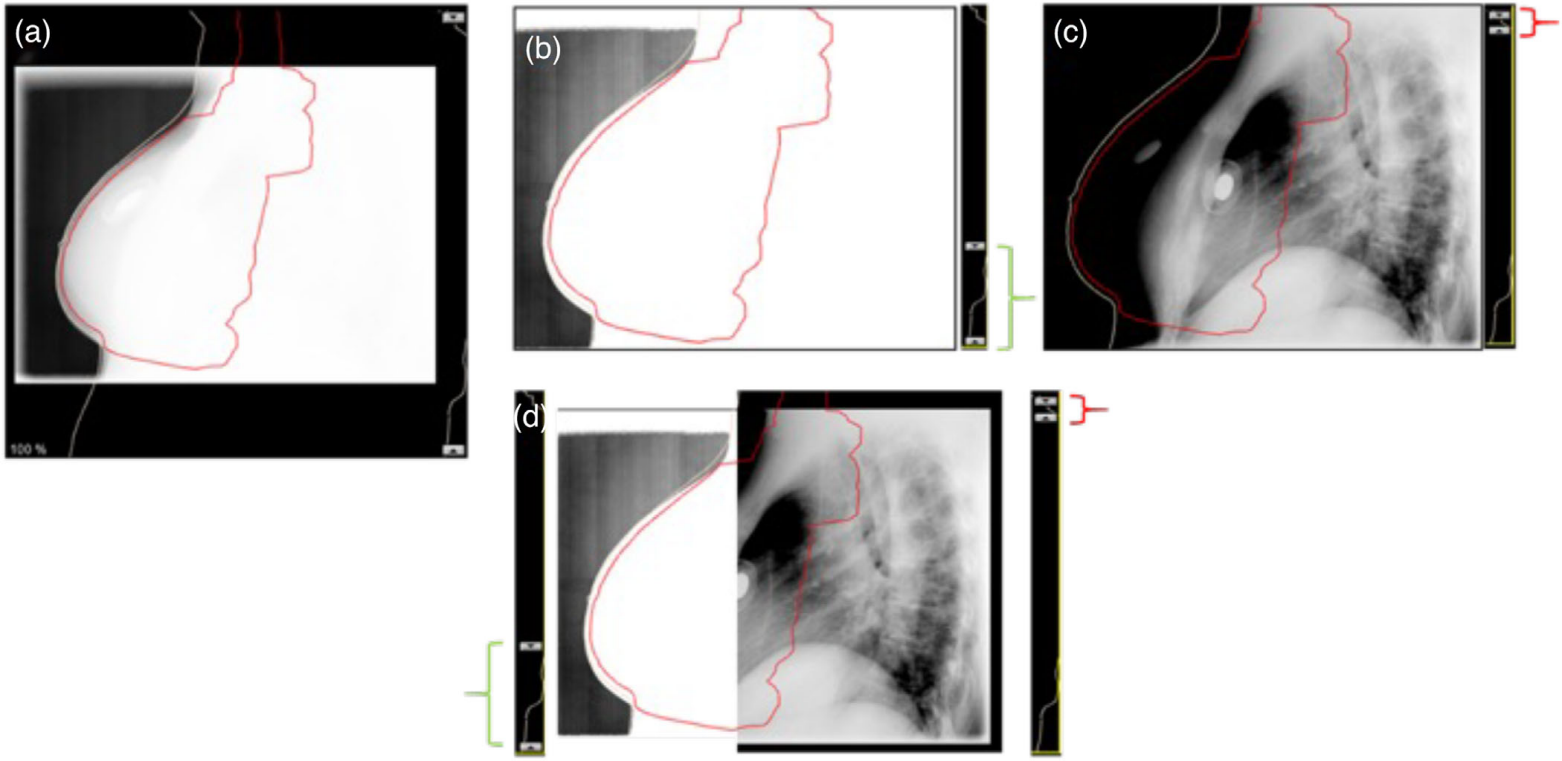
Clinical scenario in radiation therapy that motivated this study. (a) Breast lateral radiograph displayed at full W/L settings, where the outline of the breast tissue is faintly visible, but bony structures are nearly imperceptible. (b) Adjusting the W/L settings enhances visualization of breast tissue but reduces visibility of bony structures. (c) A different W/L setting improves bone visualization but diminishes the clarity of soft tissue structures. (d) Example of an approach where separate ROIs are independently adjusted, allowing both the breast tissue outline and spine to be simultaneously visible.

This challenge became evident in a clinical scenario where a breast lateral radiograph was used during radiation therapy setup, as seen in Figure 1. Under default W/L settings, breast tissue was visible, but bone structures were nearly imperceptible. Adjusting the display to emphasize bone contrast rendered the soft tissue less discernible. This highlighted a fundamental issue—image display settings alone cannot fully resolve differences in *subject contrast*, which is dependent on x‐ray energy and tissue attenuation properties.^4^, ^5^, ^6^ What options exist to improve overall contrast visualization across different structures without compromising the visibility of others? Clinical radiography does not typically have access to dual‐energy (DE) capabilities due to hardware limitations and increased radiation dose considerations.^7^, ^8^ This study evaluates whether machine learning‐based energy translation could provide a tool for conventional radiographs, enabling subject‐based contrast enhancement without requiring DE acquisition.

**FIGURE 2:**
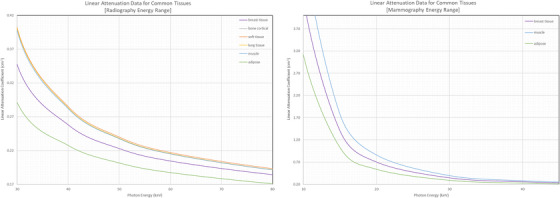
Linear attenuation data for common tissues in the radiography energy range (left) and mammography energy range (right). Greater subject contrast, defined as the difference in linear attenuation between tissues, is observed at lower x‐ray energies due to the increased separation of attenuation coefficients. For polyenergetic x‐ray sources, the observed contrast is representative of the mean effective energy of the x‐ray spectrum, as the beam consists of a range of photon energies rather than a single monoenergetic value.

The goal is to train a model to translate radiographic images between different energy domains. This approach leverages dual‐energy CT (DECT) datasets and virtual monoenergetic reconstructions to generate digitally reconstructed radiographs (DRRs) that capture varying contrast levels depending on polyenergetic and monoenergetic energies.^9^, ^10^, ^11^ As shown in Figure 2, the attenuation properties of common tissues vary significantly across energy ranges, with greater spectral separation and subject contrast achieved at lower photon energies. The data from the National Institute of Standards and Technology (NIST) X‐ray Mass Attenuation Coefficients database was used to generate these attenuation curves.^12^ By translating a polyenergetic image to another energy level for the entire image or within a specific ROI, our framework aims to improve diagnostic utility and address limitations of single‐energy radiography.

In conventional radiography, displayed image quality is often optimized post‐acquisition through adjustments like window and level, and other post‐processing techniques. While effective, these methods primarily enhance *display contrast* rather than capture intrinsic *subject contrast* differences due to the attenuation properties of tissues at different energy levels. Current software tools often lack the flexibility to independently adjust window and level settings for sub‐regions within an image, limiting contrast adjustments to the image as a whole. By enabling energy‐level translation across the full image or specific ROIs, this framework could offer a unique tool to provide enhanced diagnostic information without additional dose. To achieve this, we propose using a Generative Adversarial Network (GAN), which is well‐suited for radiographic translation tasks given the availability of paired datasets.^13^, ^14^, ^15^, ^16^, ^17^ While dual‐energy radiography (DER) datasets are limited, DECT‐derived DRRs provide a viable alternative to demonstrate feasibility, allowing for the synthesis of images across different energy levels.

DER provides a solution to varying subject contrast by acquiring images at two different x‐ray energies, enabling computational separation of materials (e.g., bone vs. soft tissue), and providing images acquired at two different x‐ray energies. However, these techniques require specialized hardware and expose patients to additional radiation, limiting their widespread adoption. They also have limitations, such as increased noise at lower energy levels and potential motion artifacts from sequential acquisitions.^7^, ^8^, ^18^

An alternative approach is a software‐driven solution in which a machine learning model could be trained to retrospectively adjust the appearance of an x‐ray image as if it had been acquired at a different energy. This concept somewhat mirrors DECT virtual monoenergetic imaging, where post‐processing algorithms generate images at arbitrary energy levels. Instead of relying on hardware modifications, this would allow for digital manipulation of contrast across an entire image or within specific ROIs, making it possible to enhance the visibility of different tissues without requiring multiple exposures.

To evaluate this concept, we propose a machine learning‐based framework for digital energy modulation, enabling translation between different x‐ray energy domains. Ideally, this framework would be trained using paired DER datasets; however, such datasets are not publicly available. To overcome this limitation, we leveraged DECT datasets to generate DRRs as paired training data. These DRRs allow us to investigate how polyenergetic and monoenergetic images can be used to train and assess a model's ability to perform energy translation. In this context, “polyenergetic” refers to images acquired using a broad spectrum of x‐ray energies, as is typical in clinical radiography and CT. In DECT, two distinct polyenergetic acquisitions are obtained, with the specific energy spectra dependent on the DECT technology used. These acquisitions provide energy‐dependent contrast differences that can be leveraged for material decomposition and virtual monoenergetic imaging. We refer to these as “low‐energy” and “high‐energy” polyenergetic images, reflecting their respective acquisition settings. These differ from monoenergetic images, which are computationally reconstructed at discrete energy levels (e.g., 60 keV, 80 keV, etc.).

Research specific to radiographic energy translation is limited. The closest‐related works involve translating chest x‐rays into bone/tissue‐only images using DER techniques^19^, ^20^, ^21^, ^22^, ^23^ and translating megavoltage digital radiographs into kilovoltage DRRs for easier patient alignment in radiation therapy.^24^, ^25^ The scarcity of research in this area may stem from the difficulty in acquiring the datasets needed to train models capable of accomplishing these image translation tasks.

We present a proof of concept for digital energy modulation using deep learning. By demonstrating this framework with DECT‐derived DRRs, we take an initial step toward software‐based contrast optimization, expanding the possibilities for image contrast adjustments in clinical imaging without requiring hardware modifications.

## 2 METHODS

**FIGURE 3:**
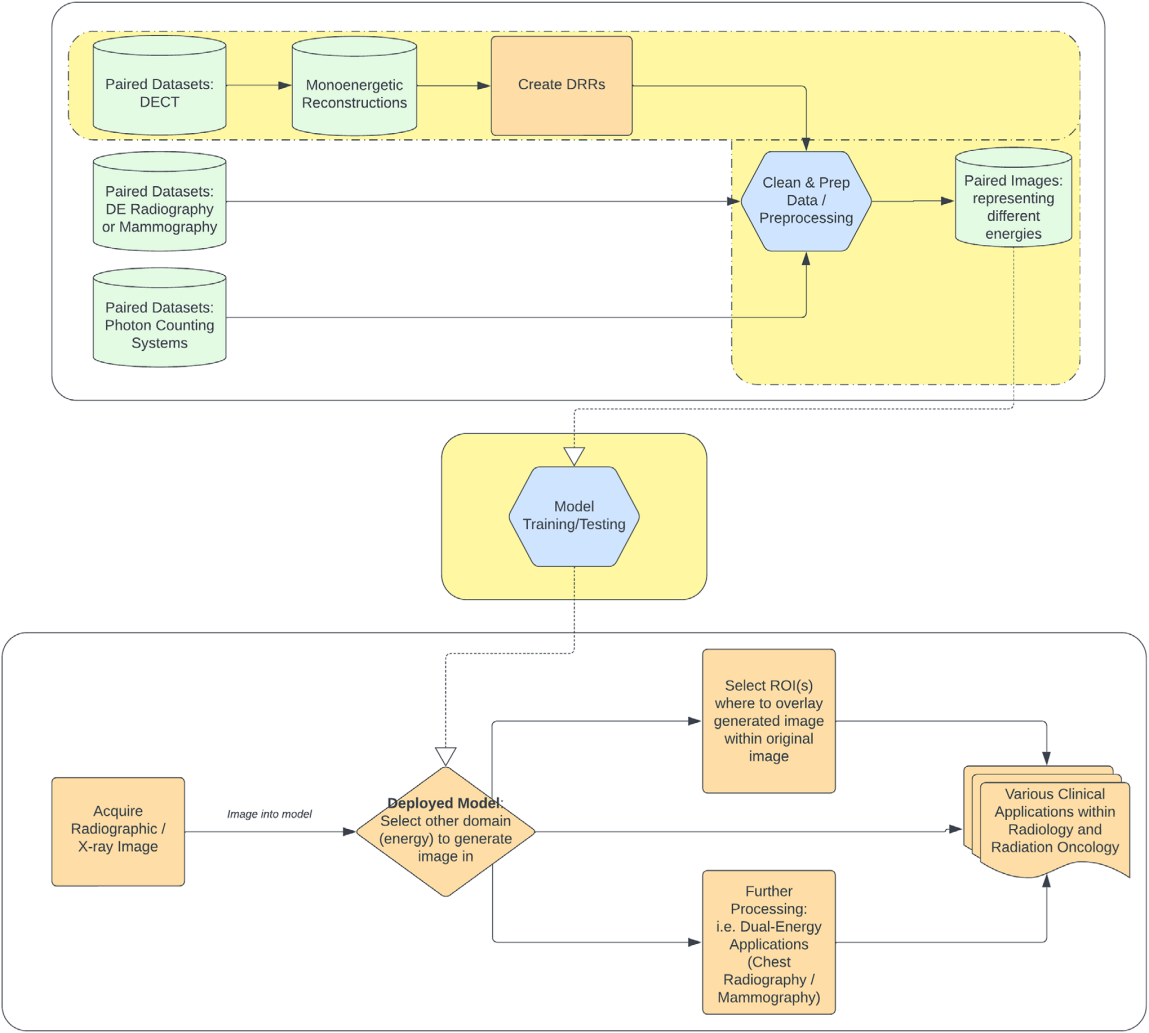
Flowchart representing an overview of the framework, including data sources and processing, model training/testing, and deployment for various potential clinical uses. The areas highlighted in yellow represent the areas this work focused on.

Figure 3 outlines the overall workflow of the proposed framework. It provides a high‐level overview of the concept, spanning potential data sources to potential clinical applications. Areas highlighted in yellow indicate the specific components of the framework investigated and will be detailed in the following sections.

### 2.1 Datasets

The study was given Institutional Review Board (IRB) exempt status. The IRB approved the retrospective usage of DECT datasets from 500 patients at the institution. At the time of data collection, the institution only used DE technology for pulmonary embolism cases. The DE studies were performed on Siemens CT scanners using dual‐source technology or through a single source using Siemens TwinBeam technology. The dual‐source technology uses two independent x‐ray sources operated at two different voltages, with the higher energy spectrum using a tin filter for even better spectral separation. Siemens TwinBeam technology uses a single source operating at a given voltage (120 or 140 kVp) filtered by either tin (high energy) or gold (low energy) to generate spectral separation.^26^

Of the 500 patients, 428 were acquired with the dual‐source CT and 72 with the CT using DE TwinBeam. A more detailed breakdown of the initial CT datasets and DRR datasets can be found in Table 1.

**TABLE 1:** CT and DRR dataset information.

CT Dataset information			DRR dataset information	
**Parameter**		**Value(s)/ information**	**Parameter**	**Value(s)/ information**
Dual‐energy acquisition type	Dual source (ds)	*428 [Scan FOV: 50 cm (A); 33 cm (B)]*	Energies represented	*Polyenergetic (kVp): 100, 120(Au,Sn), 140(Au,Sn), 140(Sn)*
				*Monoenergetic (keV): 60, 80, 100, 120*
	TwinBeam (tb)	*72 [Scan FOV: 50 cm]*		
Energies represented		*Polyenergetic (kVp): 100, 120(Au,Sn), 140(Au,Sn), 140(Sn)* *Monoenergetic (keV): 60, 80, 100, 120*	DRR calculation method	Parallel Ray Algorithm
			Source‐to‐detector distance (mm)	∞
Recon kernel		*Bf37f, Br34f, Qr40f, Qr40f∖3*	Pixel size x (mm)	1.0
Recon FOV (mm) [min, mean ± SD, max]		*[263, 341 ± 34, 492]*	Pixel size y (mm)	1.0
Isocenter (mm)		*535, 595*	Image size – rows (pixels) [min, mean ± SD, max]	*[512, 755 ± 85, 1105]*
Source‐to‐detector distance (mm)		*976, 1085.6*	Image size – columns (pixels) [min, mean ± SD, max]	*[512, 512 ± 0, 512]*
Pixel size (mm) [min, mean ± SD, max]		*[0.51, 0.67 ± 0.07, 0.96]*		
Slice thickness (mm)		*0.6*		
Slice interval (mm)		*0.4*		

The DECT datasets were processed on Siemens syngo.via which is a multimodality reading solution built on a client‐server platform. It has many packages available for different imaging applications. One of the applications makes use of DECT datasets where virtual monoenergetic images (40–190 keV possible) or material‐specific images can be created. Monoenergetic reconstructions were created at 60, 80, 100, and 120 keV. The 6 CT datasets (high polyenergetic, low polyenergetic, 60, 80, 100, and 120 keV) per patient were then anonymized and exported for further processing. The high polyenergetic (*polyhigh)* dataset includes the dual source 140 kVp and TwinBeam 120/140 kVp (Sn) data. The low polyenergetic (*polylow)* dataset includes the dual source 100 kVp and TwinBeam 120/140 kVp (Au) data. These datasets were grouped based on their corresponding high‐ and low‐energy acquisitions rather than by scanner type. This grouping naturally followed from how the DECT datasets were collected, as both dual‐source and TwinBeam acquisitions contained distinct high‐ and low‐energy components.

The anonymized datasets were then uploaded into MIM Maestro (MIM Software Inc, versions 7.1.5 and 7.2.7),^27^ which is a software package with a comprehensive set of radiation oncology tools. MIM was used for its ability to create DRRs and workflows for streamlined processing. DRRs generated on‐the‐fly (outside of treatment plans) are created using parallel rays, where the source‐to‐image distance is infinite as opposed to a virtual x‐ray source with an image plane at a set distance. The pixel sizes (x,y) are hard‐coded to be 1 mm. DRRs were generated for each dataset in 15° increments from 0° to 90°. This created 3500 images in each energy domain (six total domains: two polyenergetic, four monoenergetic).

### 2.2 Datasets preparation

Until this point, datasets were still in DICOM format. The remaining image processing and data analysis were performed in MATLAB. Due to requirements of the open‐source iteration of *Pix2Pix* used on Git Hub, the images were converted from DICOM to portable network graphics format (png) and from 16‐bit to 8‐bit and RGB format. In order to go from grayscale to RGB images, all channels were set to the same pixel value for a given pixel location. The images were ultimately resized to *256 ×* *256*. This resizing was advantageous as it ensured the image matrix primarily focused on the patient anatomy of interest. In contrast, larger matrices, such as those sized *512 ×* *512*, often included significant portions of irrelevant background outside the anatomy. On average, the proportion of pixels corresponding to the background in *512 ×* *512* images was 56.8%, compared to just 2.7% in the resized *256 ×* *256* images. From Table 1, the maximum and minimum sizes of the DRRs were *1105 ×* *512* and *512 ×* *512* respectively [row, column]. The images were first cropped and centered to *512 ×* *512*. From there, they were cropped and centered again to *256 ×* *256*. 16‐bit to 8‐bit conversion can be expressed as follows:

(1) $$DRR_{16-bit,new}\left(x,y\right)=DRR_{16-bit,orig}\;\left(x,y\right)+1$$

(2) $$DRR_{NORM}\left(x,y\right)=\frac{DRR_{16-bit,new}\left(x,y\right)}{max[DRR_{16-bit,new}\left(x,y\right)]}\;$$

(3) $$DRR_{8-bit}\left(x,y\right)=uint8\left(\left(DRR_{NORM}\left(x,y\right)\times 2^{8}\right)-1\right)$$

In this approach, normalization is performed using the maximum value of the image to scale pixel intensities. This method is appropriate when pixel values occupy only a small fraction of the available range, allowing contrast to be stretched across the full 8‐bit scale while preserving relative intensity. By scaling to the maximum, our method maintains an implied offset from zero within the data, ensuring that the original intensity distribution is preserved without unnecessary compression, and that the highest pixel value reaches the 8‐bit maximum.

### 2.3 Machine learning model and training strategy

#### 2.3.1 Model architecture and training setup

A conditional generative adversarial network^28^ (cGAN), based on Pix2Pix^29^ architecture, was used for image‐to‐image translation between different x‐ray energy domains. The goal was to evaluate the feasibility of digital energy modulation rather than to develop a novel deep‐learning model. A 256 × 256 U‐Net architecture was used for the generator, while a 70 × 70 PatchGAN was implemented for the discriminator. A summary of both networks can be found in Table 2. The models were trained using paired datasets of DRRs from DECT datasets. The Adam optimizer (*β*_1_ = 0.5, *β*_2_ = 0.999) was applied, with an initial learning rate of 0.0002 and a linear decay policy after a set number of epochs.

**TABLE 2:** Pix2Pix network summaries for generator and discriminator.

Generator network (UNet–256) summary			
Layer (type) and layer numbers			Output shape
Conv2d, LeakyReLU	(1‐2)	Unet—Encoder	[64,128,128]
Conv2d, InstanceNorm2d, LeakyReLU	(3‐5)		[128,64,64]
	(6‐8)		[256,32,32]
	(9‐11)		[512,16,16]
	(12‐14)		[512,8,8]
	(15‐17)		[512,4,4]
	(18‐20)		[512,2,2]
Conv2d, ReLU	(21‐22)	Unet—Decoder	[512,1,1]
ConvTranspose2d,InstanceNorm2d	(23‐24)		[512,2,2]
UnetSkipConnectionBlock, ReLU	(25‐26)		[1024,2,2]
ConvTranspose2d,InstanceNorm2d, Dropout	(27‐29)		[512,4,4]
UnetSkipConnectionBlock, ReLU	(30‐31)		[1024,4,4]
ConvTranspose2d,InstanceNorm2d, Dropout	(32‐34)		[512,8,8]
UnetSkipConnectionBlock, ReLU	(35‐36)		[1024,8,8]
ConvTranspose2d,InstanceNorm2d, Dropout	(37‐39)		[512,16,16]
UnetSkipConnectionBlock, ReLU	(40‐41)		[1024,16,16]
ConvTranspose2d,InstanceNorm2d	(42‐43)		[256,32,32]
UnetSkipConnectionBlock, ReLU	(44‐55)		[512,32,32]
ConvTranspose2d,InstanceNorm2d	(46‐47)		[128,64,64]
UnetSkipConnectionBlock, ReLU	(48‐49)		[256,64,64]
ConvTranspose2d,InstanceNorm2d	(50‐51)		[64,128,128]
UnetSkipConnectionBlock, ReLU	(52‐53)		[128,128,128]
ConvTranspose2d, Tanh, UnetSkipConnectionBlock	(54‐56)		[3,256,256]

**Discriminator network (PatchGAN 70 × 70) summary**			
**Layer (type) and layer numbers**			**Output shape**
Conv2d, LeakyReLU	(1‐2)		[64,128,128]
Conv2d, InstanceNorm2d, LeakyReLU	(3‐5)		[128,64,64]
	(6‐8)		[256,32,32]
	(9‐11)		[512,31,31]
Conv2d	(12)		[1,30,30]

Abbreviations: Conv2d, 2D convolution; ConvTranspose2d, transposed convolution; Dropout, regularization; InstanceNorm2d, instance normalization; LeakyReLU, leaky rectified linear unit; ReLU, rectified linear unit; Tanh, hyperbolic tangent activation function; and UnetSkipConnectionBlock, UNet skip connection block.

Training was conducted on the Huff cluster at Virginia Commonwealth University's (VCU) High Performance Research Computing Core (HPRC) using NVIDIA V100 (32GB) or A100 (80GB) GPUs. The training parameters included a batch size of eight, 200 initial epochs followed by 100 epochs with a linear decay learning rate, and an L1 loss combined with adversarial loss as defined in the original Pix2Pix paper.^29^ Instance normalization was used, and preprocessing steps involved resizing and cropping images to 256 × 256. Checkpoints were saved every five epochs for evaluation. The average training time for experiments utilizing the full datasets was ∼2.8 h per model, depending on GPU availability, while experiments with reduced dataset sizes required less training time.

#### 2.3.2 Model optimization and hyperparameter tuning

Model optimization was performed through iterative hyperparameter tuning to identify the best‐performing configuration, designated as Pix2Pix—Opt_8. The optimization process was structured into three stages. First, individual hyperparameters such as batch size, learning rate, and normalization techniques were varied while holding other parameters constant. Second, batch size, training schedule, and normalization technique were fixed, while learning rates and GAN modes were tested. Lastly, extended training schedules were evaluated with fixed batch size, learning rate, and normalization, while varying GAN architectures.

Different GAN modes were evaluated to assess training stability and image quality. Specifically, we compared the Vanilla GAN,^28^ which applies the original cross‐entropy objective, with the Least Squares GAN^30^ (LS‐GAN), which replaces the cross‐entropy with a least‐squares loss to improve convergence and stability. While both models achieved comparable results, the LS‐GAN yielded slightly superior performance across all metrics and was selected for final model training.

The loss functions (4‐5) and generator objective (6) for our Pix2Pix model using LS‐GAN are defined as follows. Let x be the input image (e.g., high‐energy image), y be the ground truth target image (e.g., low‐energy image), *G(x)* be the generated image, and *D(x,∙)* be the discriminator's output given the conditional input:

(4) $$\begin{array}{cc} & \mathrm{Adversarial}\;\mathrm{loss}\;\left(\mathrm{LS}-\mathrm{GAN}\right):\;LGAN\;\left(G,D\right)\\ & \;=Ex,y\left[{\left(D\left(x,y\right)-1\right)}^{2}\right]+Ex\left[{\left(D\left(x,G\left(x\right)\right)\right)}^{2}\right] \end{array}$$

(5) $$\begin{array}{cc} & \mathrm{Reconstruction}\;\mathrm{loss}\;\left(L1\;\mathrm{loss}\right):\;LL1\;\left(G\right)\\ & \;=Ex,y\left[∥y-G\left(x\right){∥}_{1}\right] \end{array}$$

(6) $$\begin{array}{cc} & \mathrm{Final}\;\mathrm{generator}\;\mathrm{objective}:\;\;G^{*}\\ & \;=\arg\;\min_{G}\max_{D}LGAN\left(G,D\right)+\lambda \;\cdot \;LL1\left(G\right)\; \end{array}$$

where *λ* = 100 balances the adversarial and reconstruction terms, consistent with the original Pix2Pix implementation.^29^

#### 2.3.3 Training strategy and dataset splits

Once optimized, the Pix2Pix—Opt_8 model was used for all subsequent training experiments. To ensure robustness, *k*‐fold cross‐validation (*k* = 5) was employed in the various experiments using MATLAB's *cvpartition* function, with the dataset partitioned into five folds with an 80/20 training/testing split.

Several dataset manipulations were investigated. Table 3 summarizes the different model translations and training options. Baseline training was conducted using full dataset cross‐validation, performing energy translation across polyenergetic and monoenergetic domains. In each experiment, the model was trained to translate from Dataset A (input image) to Dataset B (target image), where the specific source and target energy domains (e.g., polyhigh to 60 keV) are defined per translation task in Table 3.

**TABLE 3:** Model training and testing information for the various dataset manipulations investigated.

	Model	Train/Test (# images)	Data split	Dataset A	Dataset B
*K‐fold Cross Validation*	Pix2Pix—Opt_8	2800/700	split_data_k[1‐5]	polyhigh	polylow; [60,80,100,120] keV
	Pix2Pix—Opt_8	2800/700	split_data_k[1‐5]	polylow	polyhigh; [60,80,100,120] keV
	Pix2Pix—Opt_8	∼2800/700	split_data_k[1‐5]	PH woTB	PL; [60,80,100,120] keV—woTB
	Pix2Pix—Opt_8	∼2800/700	split_data_k[1‐5]	PL woTB	PH; [60,80,100,120] keV—woTB
*DSR*	Pix2Pix—Opt_8	1400/700	split_data_k[1‐5]	polyhigh	polylow
	Pix2Pix—Opt_8	700/700	split_data_k[1‐5]	polyhigh	polylow
*PS (AP/LAT)*	Pix2Pix—Opt_8	400/100	split_data_k[1‐5]_AP	polyhigh	polylow
	Pix2Pix—Opt_8	400/100	split_data_k[1‐5]_LAT	polyhigh	polylow

Abbreviations: DSR, data size reduction; PH, polyhigh; PL, polylow; PS (AP/LAT), Projection Specific (Anterior‐Posterior / Lateral); and woTB, without TwinBeam data.

To assess the effect of dataset size, dataset size reduction (DSR) was implemented by reducing the training dataset to 50% and 25% of its original size while maintaining a 20% test set. Projection‐specific training was also explored by restricting training datasets to anterior‐posterior (AP) and lateral (LAT) projections, which resulted in a significantly reduced dataset size, with training data constituting 11.4% and test data 2.85% of the original dataset.

To assess whether DECT acquisition technology influenced translation performance, models were trained both on the full dataset and on a subset where TwinBeam data was excluded (woTB = without TwinBeam data). The woTB datasets contained only dual‐source DECT images while preserving the same randomized cross‐validation splits as the full dataset. This ensured that any observed differences in model performance were attributable to the absence of TwinBeam data rather than differences in dataset partitioning splits from the full dataset.

### 2.4 Quantitative analysis

To evaluate model performance, four standard image quality metrics were used: peak signal‐to‐noise ratio (PSNR), structural similarity index measure (SSIM), mean squared error (MSE), and mean absolute percentage error (MAPE). These metrics provide complementary assessments of image fidelity, capturing both pixel‐wise and perceptual differences between the generated and reference images. Each metric was computed on an image‐by‐image basis and subsequently averaged across the test dataset at each evaluation epoch. These metrics are widely used for evaluating image quality in medical imaging and have been described in previous works.^31^, ^32^, ^33^, ^34^, ^35^, ^36^, ^37^ In addition to numerical evaluation, qualitative assessments were conducted using SSIM maps and difference maps to visually compare generated images with their corresponding reference images.

To evaluate differences in model performance under varying experimental conditions, statistical tests were conducted using paired data from *k*‐fold cross‐validation (*k* = 5). For experiments comparing models trained with and without TwinBeam data, different training dataset sizes, and projection‐specific datasets, two‐tailed paired *t*‐tests were performed for each energy translation task and evaluation metric (PSNR, SSIM, MSE, and MAPE). This test was chosen because model performance metrics were computed on the same fold‐wise partitions, making the data naturally paired. In addition, to investigate whether the similarity between input and output energy domains (i.e., SSIM between domain A and B) was associated with final model performance, correlation analysis was performed using both Pearson's correlation coefficient (*r*) and Spearman's rank correlation coefficient (*ρ*). Pearson's *r* assesses the strength of linear relationships, while Spearman's *ρ* captures monotonic associations without assuming linearity.^38^ All statistical analyses were performed using MATLAB's built‐in functions. A significance threshold of *p* < 0.05 was used to determine statistical significance. Results with 0.05 ≤ *p* < 0.1 were considered marginally significant, and those with *p* ≥ 0.1 were considered not statistically significant. For visualization, color‐coded heatmaps were generated to summarize *p*‐value comparisons across experimental conditions.

#### 2.4.1 Image quality metrics

1. PSNR measures the logarithmic ratio between the maximum possible pixel intensity and the mean squared error (MSE), with higher values indicating better quality.
2. SSIM evaluates perceptual image similarity, incorporating luminance, contrast, and structural components. SSIM ranges from −1 to 1, with values closer to 1 indicating higher similarity between images; a value of 1 implies a perfect match.
3. MSE measures the average squared difference between pixel values in the generated and reference images. Lower MSE values are preferred, indicating reduced pixel‐wise error.
4. MAPE quantifies the average percentage error per pixel, with lower values indicating higher accuracy. Lower MAPE values are desirable, reflecting minimal percentage‐based deviation between images.

All calculations were implemented using built‐in MATLAB functions, with “omitzero” and “omitnan” flags applied to MAPE to prevent division errors where the reference pixel value was zero.

### 2.5 Model testing information

Model testing was conducted on the Huff cluster at VCU's HPRC, utilizing the same NVIDIA V100 (32GB) or A100 (80GB) GPUs as the training phase whenever possible. In cases where these GPUs were unavailable, standard compute nodes equipped with AMD 2x Epyc2 64‐core CPUs or AMD 2x Epyc3 64‐core CPUs were used.

The trained models were used to generate test images, which were then exported to a secure, restricted network location for evaluation. MATLAB scripts were used to compute the PSNR, SSIM, MSE, and MAPE for all generated images, providing an objective assessment of the model's performance.

## 3 RESULTS

### 3.1 Evaluation metrics

In the following sections, higher values represent better model performance for PSNR and SSIM, while lower values are better for MSE and MAPE. For experiments using 5‐fold cross‐validation, results in tables are reported as the mean ± standard deviation across the average metric values from each model fold.

### 3.2 Cross‐validation and TwinBeam data exclusion

**FIGURE 4:**
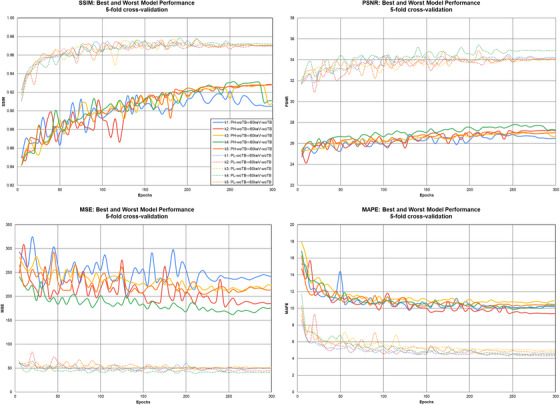
Best‐performing and worst‐performing cross‐validation trained models on two different energy domain translations using the four image quality metrics as a function of epochs.

Table 4 shows the results for the cross‐validation models with and without TwinBeam data. Figure 4 shows the polyhigh‐woTB → 60 keV‐woTB and polylow‐woTB → 60 keV‐woTB results for the different folds across all epochs. These were the worst and best‐performing energy domain translations, respectively. Figure 4 highlights the variability in results depending on the data splits used. It also shows when the model performance metrics start to stabilize and level off with minimal improvement.

**TABLE 4:** Quantitative evaluation of models trained using 5‐fold cross‐validation with and without TwinBeam data.

Epoch	Data split	Dataset A	Dataset B	PSNR		SSIM		MSE		MAPE	
300	split_data_k[1‐5]	polyhigh	60 keV	27.26	± 0.43	0.920	± 0.012	196.5	± 19.2	9.9	± 0.4
		polyhigh‐woTB	60 keV‐woTB	26.95	± 0.34	0.916	± 0.011	208.0	± 26.7	10.2	± 0.5
		polyhigh	80 keV	31.30	± 0.19	0.959	± 0.001	83.1	± 5.9	5.7	± 0.2
		polyhigh‐woTB	80 keV‐woTB	31.22	± 0.30	0.961	± 0.001	83.5	± 6.7	5.7	± 0.2
		polyhigh	100 keV	29.51	± 0.32	*0.963*	± 0.002	137.6	± 14.9	7.2	± 0.4
		polyhigh‐woTB	100 keV‐woTB	30.16	± 0.39	0.966	± 0.002	116.4	± 14.9	6.4	± 0.3
		polyhigh	120 keV	28.77	± 0.36	0.957	± 0.001	161.3	± 18.6	7.7	± 0.3
		polyhigh‐woTB	120 keV‐woTB	29.41	± 0.42	0.960	± 0.001	131.6	± 14.6	6.7	± 0.2
		polyhigh	polylow	27.51	± 0.54	0.923	± 0.004	186.7	± 23.9	9.3	± 0.3
		polyhigh‐woTB	polylow‐woTB	27.67	± 0.42	0.925	± 0.003	177.6	± 20.8	9.2	± 0.3
		polylow	60 keV	*33.21*	± 0.38	0.961	± 0.002	*67.3*	± 7.1	*5.3*	± 0.3
		polylow‐woTB	60 keV‐woTB	*34.25*	± 0.38	*0.971*	± 0.001	*47.5*	± 4.7	*4.6*	± 0.3
		polylow	80 keV	30.40	± 0.39	0.963	± 0.002	132.6	± 17.7	7.3	± 0.6
		polylow‐woTB	80 keV‐woTB	31.17	± 0.24	0.965	± 0.002	98.2	± 10.7	6.3	± 0.4
		polylow	100 keV	28.05	± 0.58	0.947	± 0.007	241.3	± 44.5	9.4	± 0.9
		polylow‐woTB	100 keV‐woTB	29.18	± 0.32	0.948	± 0.001	144.3	± 19.2	7.3	± 0.6
		polylow	120 keV	27.47	± 0.36	0.943	± 0.008	282.4	± 46.7	10.1	± 0.9
		polylow‐woTB	120 keV‐woTB	28.58	± 0.27	0.940	± 0.000	164.2	± 17.5	7.9	± 0.5
		polylow	polyhigh	27.24	± 0.42	0.935	± 0.007	202.0	± 26.3	10.0	± 0.8
		polylow‐woTB	polyhigh‐woTB	27.57	± 0.22	0.935	± 0.003	181.2	± 17.2	9.7	± 0.8

Abbreviation: *woTB, without TwinBeam data*.

*Note*: *The best‐performing model results for both with and without TwinBeam data for a given metric are bolded, underlined, and italicized*.

To ensure that model performance comparisons were not biased by any particular training/test partitioning, all energy translation tasks were evaluated using *k*‐fold cross‐validation (*k* = 5). This approach provided a consistent framework for comparing models trained with and without TwinBeam data. Despite a reduction in dataset size, models trained without TwinBeam data generally demonstrated equivalent or improved performance across the majority of evaluation metrics.

Paired *t*‐tests were performed across ten energy translation tasks and four evaluation metrics (PSNR, SSIM, MAPE, and MSE), for a total of 40 comparisons. Statistically significant improvements (*p* < 0.05) were observed in 25 of these comparisons, with an additional one showing marginal significance (0.05 ≤ *p* < 0.1). In terms of average performance (across all five folds), 31 of the 40 comparisons showed improvements for models trained without TwinBeam data, as indicated by upward arrows in Table 5. The most consistent improvements—both statistically and in average metric values—were observed for polyhigh → 100 keV, polyhigh → 120 keV, and polylow → 60 keV, all of which showed significant gains across all four metrics.

**TABLE 5:** *P*‐values from paired *t*‐tests comparing model performance with and without TwinBeam data across energy translations.

With vs. without TwinBeam data	PSNR *p*‐value	SSIM *p*‐value	MAPE *p*‐value	MSE *p*‐value
polyhigh → 60 keV	↓ 0.1379	↓ 0.6728	↓ 0.0442	↓ 0.0903
polyhigh → 80 keV	↓ 0.2551	↑ 0.0271	↓ 0.3215	0.8366
polyhigh → 100 keV	↑ 0.0011	↑ 0.0074	↑ 0.0006	↑ 0.0005
polyhigh → 120 keV	↑ 0.0016	↑ 0.0010	↑ 0.0007	↑ 0.0007
polyhigh → polylow	↑ 0.2010	↑ 0.1910	↑ 0.1359	↑ 0.0258
polylow → 60 keV	↑ 0.0000	↑ 0.0000	↑ 0.0003	↑ 0.0007
polylow → 80 keV	↑ 0.0147	↑ 0.2965	↑ 0.0108	↑ 0.0090
polylow → 100 keV	↑ 0.0169	↑ 0.7955	↑ 0.0042	↑ 0.0075
polylow → 120 keV	↑ 0.0063	↓ 0.4731	↑ 0.0028	↑ 0.0048
polylow → polyhigh	↑ 0.0891	0.9746	↑ 0.0331	↑ 0.0392

Green indicates *p* < 0.05 (statistically significant), yellow indicates 0.05 ≤ *p* < 0.1 (marginal), and red indicates *p* ≥ 0.1 (not significant). Up (↑) and down (↓) arrows preceding a value denote an increase or decrease, respectively, in average model performance (across all 5 folds) for models trained without TwinBeam data compared to those with it.

While the polyhigh → polylow task yielded consistent improvements in average performance across three of the four metrics, only one of these improvements was statistically significant. In contrast, polyhigh → 80 keV showed mixed results, with modest improvements in SSIM but performance regressions in other metrics, none of which reached statistical significance. The polyhigh → 60 keV translation task was the only case where average performance declined across all four metrics, including one significant and one marginal *p*‐value.

These results suggest that excluding TwinBeam data—likely due to its differing spectral characteristics and acquisition methodology—can reduce domain variability and enhance model generalization. However, the extent of improvement appears to vary by energy translation task, with more pronounced benefits observed in translations to certain monoenergetic targets.

### 3.3 DSR and Projection‐Specific (AP/LAT) datasets

Table 6 shows the results for the models trained on reduced dataset sizes and projection‐specific datasets. To formally assess the impact of training dataset size, paired *t*‐tests were conducted across three training volumes: 80%, 40%, and 20%, using PSNR, SSIM, MAPE, and MSE as evaluation metrics. The corresponding *p*‐values are presented in Table 7. The results indicate that dataset size has a statistically significant impact on model performance, particularly when comparing the smallest training size (20%) to the larger sets. Reductions from 80% to 20% and from 40% to 20% yielded significant degradation across all metrics (*p* < 0.05), confirming that the lowest dataset volume consistently led to inferior performance. In contrast, the 80% versus 40% comparison resulted in fewer significant differences, with PSNR and SSIM showing marginal or significant *p*‐values. This suggests that moderate reductions in dataset size may not uniformly degrade performance, but substantial reductions (e.g., to 20%) do.

**TABLE 6:** Quantitative evaluation of models trained using reduced dataset sizes or projection‐specific datasets.

	Train/Test	Data split	Dataset A	Dataset B	Epoch	PSNR	SSIM	MSE	MAPE
*K‐fold (5) Cross Validation DSR*	80/20	split_data_k[1‐5]	*polyhigh*	*polylow*	5	26.11 ± 0.45	0.867 ± 0.002	217.8 ± 28.0	14.8 ± 1.5
	40/20					26.02 ± 0.27	0.856 ± 0.006	222.8 ± 24.1	16.2 ± 1.7
	20/20					25.69 ± 0.36	0.847 ± 0.007	239.4 ± 28.6	17.4 ± 1.6
	80/20				300	*27.51* ± 0.54	*0.923* ± 0.004	*186.7* ± 23.9	*9.3* ± 0.3
	40/20					27.27 ± 0.40	0.917 ± 0.002	193.0 ± 25.4	9.4 ± 0.3
	20/20					26.93 ± 0.41	0.910 ± 0.001	203.2 ± 25.1	10.1 ± 0.2
*K‐fold (5) Cross Validation PS (AP/LAT)*	11.4/2.85	split_data_k[1‐5]_AP	*polyhigh*	*polylow*	5	26.42 ± 0.43	0.853 ± 0.003	198.5 ± 29.6	10.0 ± 1.0
		split_data_k[1‐5]_LAT				25.55 ± 0.59	0.795 ± 0.013	259.0 ± 47.3	23.7 ± 2.9
		split_data_k[1‐5]_AP			300	*27.58* ± 0.47	*0.911* ± 0.001	*168.7* ± 25.9	*8.5* ± 0.5
		split_data_k[1‐5]_LAT				27.14 ± 0.51	0.908 ± 0.003	223.1 ± 31.9	12.3 ± 0.5

Abbreviations: *DSR, data size reduction, and PS (AP/LAT), projection specific (anterior‐posterior / lateral)*.

*Notes* The values in the Train/Test column represent the percentage distribution of images from the original dataset allocated to the training and test datasets, respectively. The best‐performing model results for the DSR and PS experiments for a given metric are bolded, underlined, and italicized.

**TABLE 7:** *P*‐values from paired *t*‐tests assessing the impact of training dataset size.

Training size comparison	PSNR *p*‐value	SSIM *p*‐value	MAPE *p*‐value	MSE *p*‐value
20% vs. 40%	↑ 0.0007	↑ 0.0007	↑ 0.0009	↑ 0.0264
20% vs. 80%	↑ 0.0057	↑ 0.0012	↑ 0.0015	↑ 0.0316
40% vs. 80%	↑ 0.0863	↑ 0.0251	↑ 0.2101	↑ 0.1634

*Energy Translation / Models*: *Polyhigh → Polylow (k = 5)*

Green indicates *p* < 0.05 (statistically significant), yellow indicates 0.05 ≤ *p* < 0.1 (marginal), and red indicates *p* ≥ 0.1 (not significant). Upward arrows (↑) denote cases where the model trained on more data outperformed the one trained on less, across all evaluation metrics.

To further evaluate the effects of dataset composition, we compared models trained exclusively on anterior‐posterior (AP) or lateral (LAT) projection images to those trained on the full dataset (80% of all projections). As shown in Table 8, AP‐specific models outperformed full‐dataset models in PSNR, MAPE, and MSE, with statistically significant improvements (*p* < 0.05) in MAPE and MSE. In contrast, LAT‐specific models performed significantly worse than full models across all metrics except PSNR. When comparing AP and LAT models directly, AP significantly outperformed LAT in MAPE and MSE, underscoring the potential impact of anatomical variability on model performance. These findings suggest that the effectiveness of projection‐specific training may depend on the anatomical detail and consistency present in a given view, with certain projections offering more favorable conditions for energy translation tasks.

**TABLE 8:** *P*‐values from paired *t*‐tests comparing projection‐specific models against the full dataset equivalent, and each other.

Projection‐Specific vs. Full	PSNR *p*‐value	SSIM *p*‐value	MAPE *p*‐value	MSE *p*‐value
AP vs. Full	↑ 0.5885	↓ 0.0055	↑ 0.0016	↑ 0.0056
LAT vs. Full	↓ 0.0956	↓ 0.0007	↓ 0.0004	↓ 0.0171
AP vs. LAT	↑ 0.1484	↑ 0.2031	↑ 0.0001	↑ 0.0088

*Energy Translation / Models*: *Polyhigh → Polylow (k = 5)*

Green indicates *p* < 0.05 (statistically significant), yellow indicates 0.05 ≤ *p* < 0.1 (marginal), and red indicates *p* ≥ 0.1 (not significant). Arrows indicate the direction of improvement: ↑ favors the first group listed, ↓ favors the second.

### 3.4 Effect of domain similarity on model performance

To investigate the impact of domain similarity on model performance, we examined the relationship between SSIM_DOMAIN_—a metric that captures the structural similarity between the input and target image domains—and our four image quality metrics. SSIM_DOMAIN_ was calculated for every energy translation task using the test datasets across all five cross‐validation folds (see Table 9 for averaged values).

**TABLE 9:** SSIM between energy domains.

Data split	Dataset A	Dataset B	SSIM_DOMAIN_	
*split_data_k[1‐5]*	polyhigh	60 keV	0.905	± 0.002
	polyhigh‐woTB	60 keV‐woTB	0.905	± 0.003
	polyhigh	80 keV	0.967	± 0.001
	polyhigh‐woTB	80 keV‐woTB	0.968	± 0.002
	polyhigh	100 keV	0.965	± 0.002
	polyhigh‐woTB	100 keV‐woTB	0.965	± 0.002
	polyhigh	120 keV	0.960	± 0.002
	polyhigh‐woTB	120 keV‐woTB	0.961	± 0.002
	polyhigh	polylow	0.931	± 0.002
	polyhigh‐woTB	polylow‐woTB	0.931	± 0.002
	polylow	60 keV	0.968	± 0.002
	polylow‐woTB	60 keV‐woTB	0.977	± 0.001
	polylow	80 keV	0.961	± 0.001
	polylow‐woTB	80 keV‐woTB	0.961	± 0.001
	polylow	100 keV	0.934	± 0.001
	polylow‐woTB	100 keV‐woTB	0.928	± 0.001
	polylow	120 keV	0.923	± 0.001
	polylow‐woTB	120 keV‐woTB	0.917	± 0.001
	polylow	polyhigh	0.931	± 0.002
	polylow‐woTB	polyhigh‐woTB	0.931	± 0.002

Abbreviation: *woTB, without TwinBeam data*.

**FIGURE 5:**
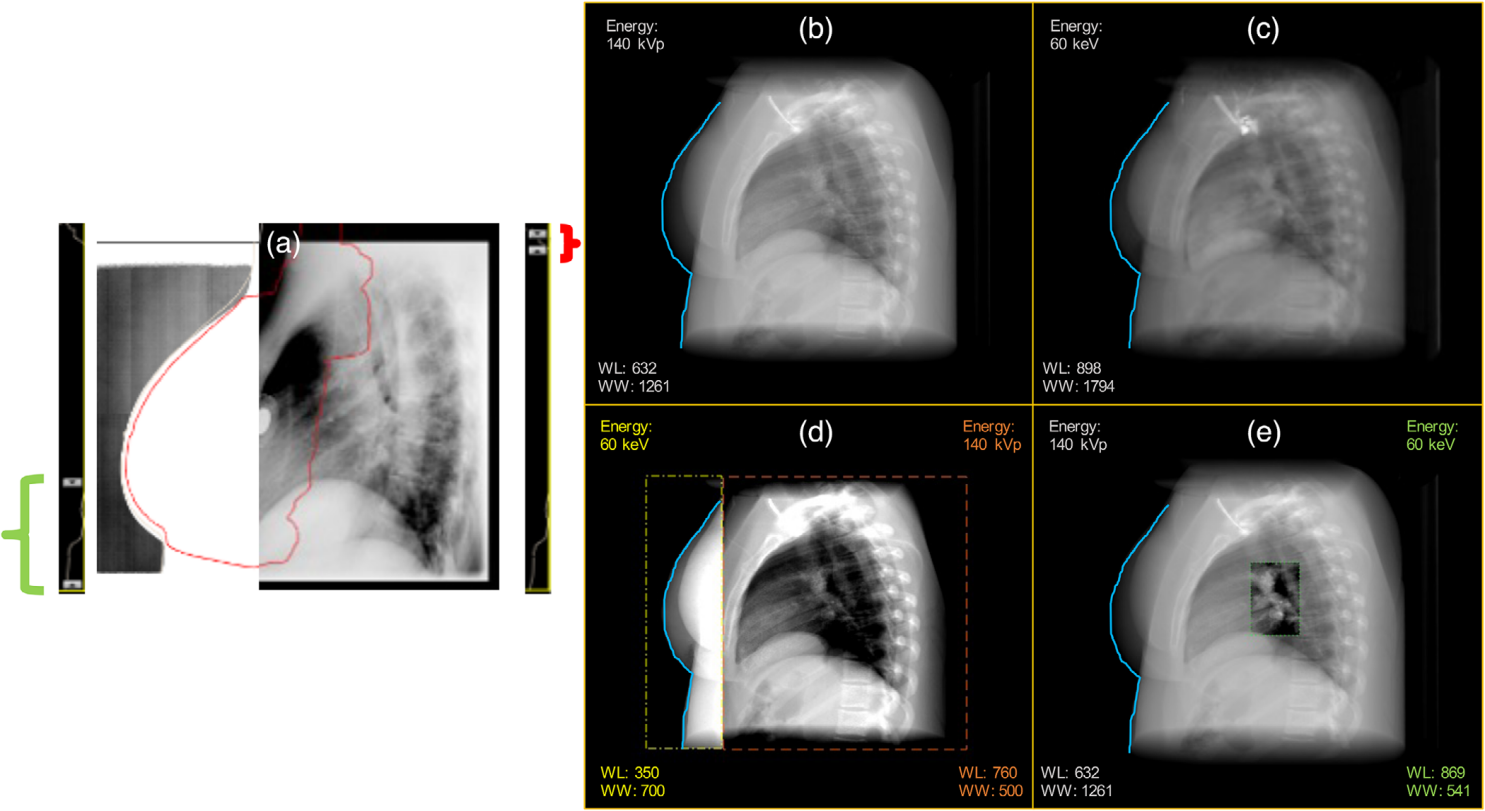
Correlation between input/output domain similarity (SSIM_DOMAIN_) and model performance metrics (PSNR, SSIM, MSE, MAPE). Each point represents the average performance metric from a fold of all models trained in the *Cross‐Validation & TwinBeam Data Exclusion* experiment. Linear regression lines highlight the overall trend.

As shown in Figure 5, a strong correlation was observed between SSIM_DOMAIN_ and model performance. Specifically, higher SSIM_DOMAIN_ values were associated with higher PSNR and SSIM, indicating improved perceptual and pixel‐wise image fidelity. Higher SSIM_DOMAIN_ also correlated with lower MSE and MAPE, suggesting more accurate and consistent predictions.

Both Pearson's (*r*) and Spearman's (*ρ*) correlation coefficients were calculated to assess linear and rank‐based relationships, respectively. All correlations were statistically significant (*p* < 0.0001) across metrics. The strongest associations were observed for SSIM (*r* = 0.881, *ρ* = 0.823) and MAPE (*r* = –0.841, *ρ* = −0.813), highlighting that increased input/output similarity leads to better model generalization and fewer image translation errors. These findings support the intuitive assumption that energy translation is easier when the underlying structural properties between domains are similar. This may also partially explain the performance variability across energy translation tasks observed in earlier experiments.

## 4 DISCUSSION

### 4.1 Implications

We have proposed a software‐based framework for digital energy modulation in projectional x‐ray imaging, which offers a potential solution to common clinical challenges involving subject contrast limitations. By enabling translation between x‐ray energy domains, this method provides a way to retrospectively modify image contrast—either across the full image or within targeted ROIs—without requiring additional exposures or hardware modifications.

**FIGURE 6:**
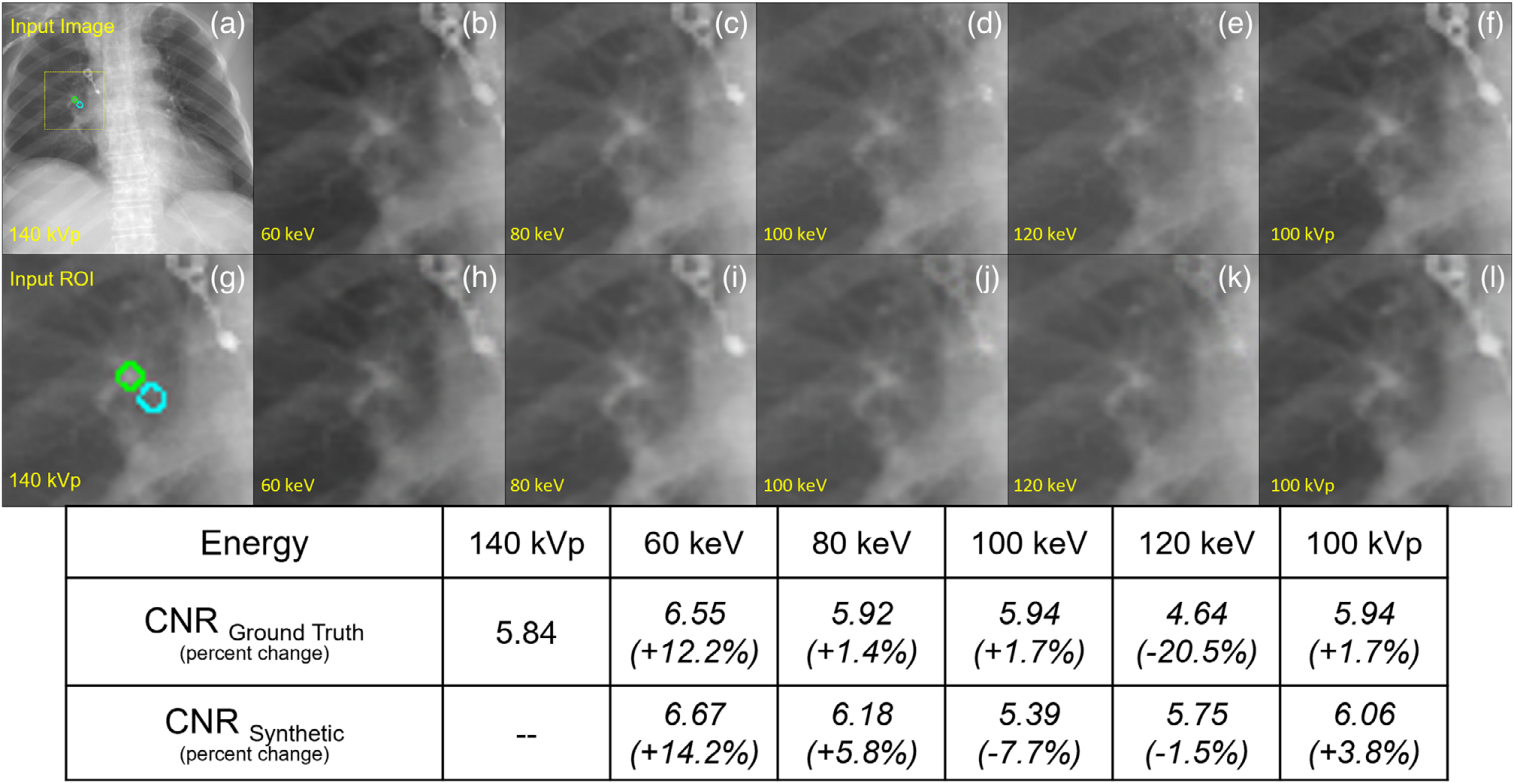
(a) The original problem in radiation therapy patient setup, where two separate window/level (W/L) settings are needed to visualize both soft tissue and bone. The problem is shown in the top row (b, c) using a lateral [90°] DRR in two energy domains. The blue outline represents breast tissue, which is not visible under full W/L settings. Panels (D) and (E) show two proposed energy modulation solutions: (d) adjusting two ROIs independently with different W/L settings and (e) applying an energy‐modulated ROI (e.g., 60 keV) for improved contrast while maintaining independent window settings.

The original clinical motivation is illustrated in Figure 6, which recreates a radiation oncology setup scenario where global W/L adjustments fail to simultaneously visualize soft tissue and bone in a lateral breast radiograph. The top row (B‐C) depicts this issue using DRRs acquired at two different energy levels. Panels (D) and (E) present two potential solutions enabled by the digital energy modulation framework: applying independent W/L adjustments to specific ROIs and using energy translation to digitally enhance contrast in selected ROIs without requiring additional acquisitions. This approach could ultimately lead to more tailored image presentation and improved workflow efficiency in clinical settings.

**FIGURE 7:**
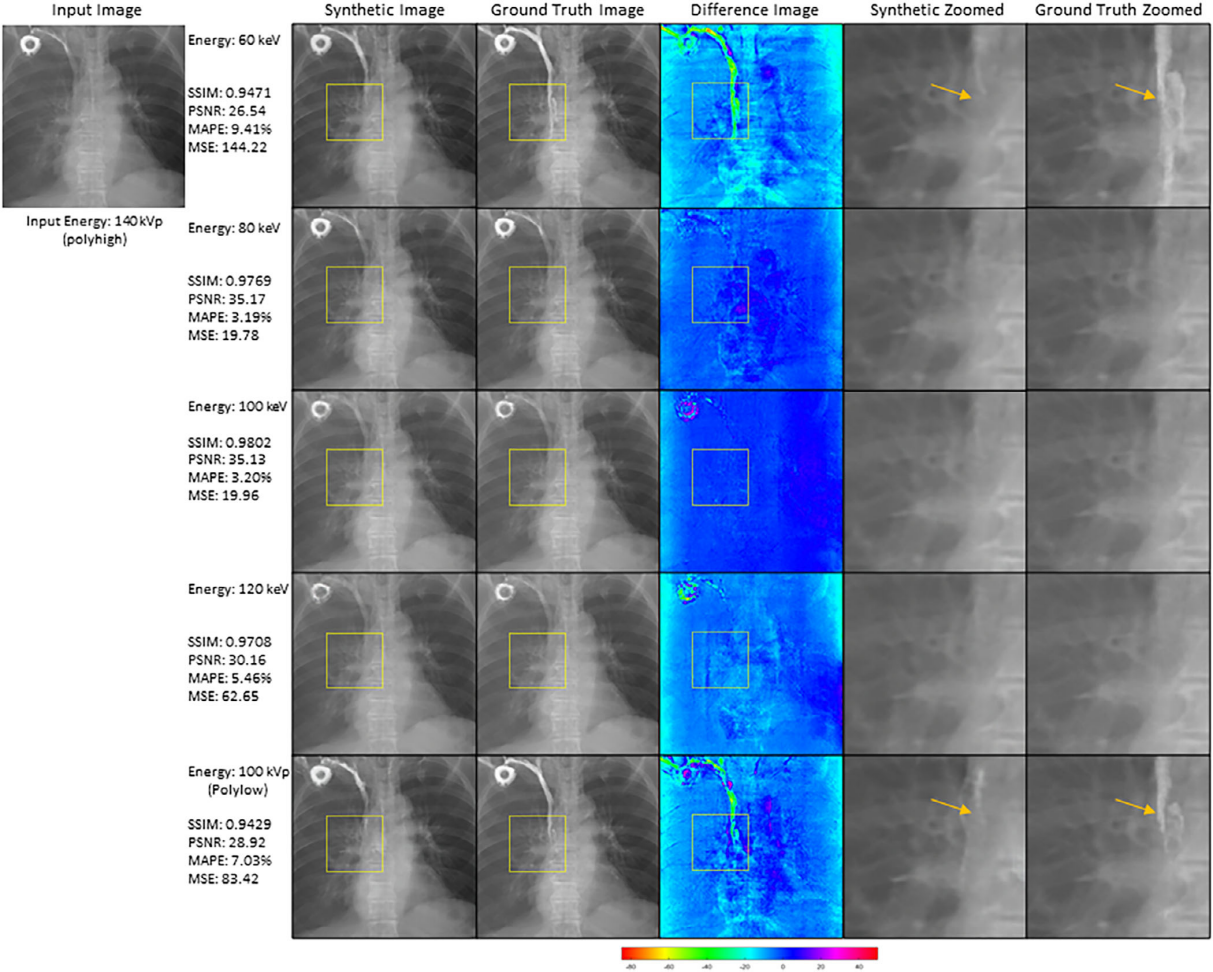
Contrast‐to‐noise ratio (CNR) comparison between ground truth (b–f) and synthetic images (h–l) across different energy levels. The input image (a) is put into the models trained on without TwinBeam data [*k* = 1]. The zoomed‐in patches show the target ROI (green ROI) and background (blue ROI) for the CNR calculation. The first row of the table provides CNR values for ground truth images, while the second row has the CNR values for the corresponding synthetic images with percent change relative to ground truth in parenthesis.

Figure 7 further supports the viability of energy domain translation as a contrast‐enhancing tool by comparing contrast‐to‐noise ratio (CNR) measurements between ground truth and synthetic images across a range of energies. As expected, CNR generally improves at lower energies. The synthetic images generated by the trained model show similar trends, although slight discrepancies are present in certain cases. This demonstrates the model's ability to preserve or even enhance energy‐dependent subject contrast, reinforcing the value of this framework for post‐acquisition contrast manipulation.

The success of energy translation models is highly dependent on the availability of high‐quality, paired datasets—ideally of the same anatomy imaged at different energy levels. Creating such datasets using conventional systems is challenging due to ethical constraints around additional radiation dose and the potential for patient motion between exposures. However, the growing adoption of photon counting detectors (PCDs) in CT offers a promising path forward. These detectors segment the x‐ray spectrum into discrete energy bins, which can be treated as paired energy datasets without requiring additional exposures. If PCDs are integrated into future projectional x‐ray systems, they could provide an ideal foundation for training energy translation models applicable to conventional digital detectors, thereby expanding access to these image enhancement tools across clinical practice.

### 4.2 Limitations

Despite demonstrating feasibility, the approach has limitations that impact generalizability. First, translating between polyenergetic domains is inherently underdetermined: multiple tissue compositions or thicknesses can produce similar attenuation at a given energy. This ambiguity makes it difficult for deep learning models to learn consistent mappings without rich, diverse training data. Moreover, biological tissues vary in density, composition, and pathology, further increasing complexity.

Another limitation lies in the use of DECT‐derived DRRs for model training. Although DRRs allow controlled experiments, they lack the resolution and noise characteristics of clinical radiographs. Monoenergetic reconstructions, while valuable for demonstrating energy‐dependent contrast differences, were vendor‐calibrated and not independently validated, potentially affecting fidelity across energy levels. While scanner calibration is beyond the scope of this study, future studies should validate these reconstructions using phantoms or Monte Carlo simulations if using monoenergetic DRRs for model training.

The chosen architecture, Pix2Pix, served as a baseline for proof‐of‐concept and was not optimized beyond standard parameters. While suitable for initial investigation, more advanced or specialized architectures may be better equipped to handle the spectral and anatomical complexity of energy domain translation. Additionally, the GitHub open‐source Pix2Pix implementation required downscaled image bit‐depth for compatibility, which deviates from clinical imaging standards. This limitation may have influenced the image quality metrics used to evaluate model performance and should be addressed in future work by incorporating higher bit‐depth datasets and models capable of supporting full clinical dynamic range.

### 4.3 Model performance and evaluation

The performance of the proposed digital energy modulation framework was evaluated across multiple experiments, each targeting different variables that could influence model output. Rather than being limited by architecture alone, performance was shaped by several key factors, including dataset composition, training volume, projection specificity, and domain similarity between the input and output energy levels.

Strong correlations were observed between SSIM_DOMAIN_ and the image quality metrics. As shown in Figure 7, models trained on domains with more similarity achieved more accurate translations. This suggests that, beyond architecture or training data size, the spectral similarity of energy domains played a role in determining model success. Paired *t*‐tests further revealed that excluding TwinBeam data, despite reducing the dataset size, improved model performance for several tasks. Similarly, models trained on reduced data volumes exhibited significant performance drops, particularly at 20% of the full dataset, confirming the importance of training set size. Lastly, projection‐specific models yielded mixed results: AP‐based models performed comparably or better than full‐dataset models, while LAT‐only models underperformed. This may reflect the relative consistency and diagnostic richness of AP views compared to the anatomical overlap and variability seen in LAT projections.

**FIGURE 8:**
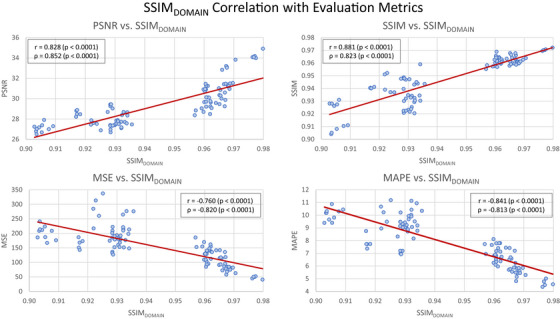
Comparison of synthetic and ground truth images across different energy levels. Column 1: Synthetic images generated by models trained without TwinBeam data [*k* = 5]. Column 2: Corresponding ground truth images. Column 3: Difference maps illustrating discrepancies. Column 4: Zoomed‐in synthetic image ROIs. Column 5: Zoomed‐in ground truth image ROIs. Arrows in the 60 keV and polylow ROIs highlight areas with contrast agent artifacts, which were more challenging for the models to replicate. SSIM, PSNR, MAPE, and MSE values are for the whole synthetic image.

Figure 8 illustrates both the strengths and limitations of the proposed energy translation framework by comparing synthetic and ground‐truth images across the five energy domains (60, 80, 100, 120 keV, and polyhigh/polylow), using results from models trained without TwinBeam data. While synthetic images often reflect expected contrast differences across energy levels, visual inspection reveals notable discrepancies in regions containing contrast agents and wires—particularly for 60 keV and polylow outputs. These deviations, highlighted in the difference maps and zoomed‐in ROIs, underscore a broader concern in GAN‐based medical imaging: the risk of omitting subtle yet clinically relevant details or introducing misleading artifacts.

To contextualize performance, Table 10 compares our best and worst models from each experiment with previously published works on related translation tasks. While no direct analog exists for our exact energy translation task, the two most similar tasks to our study are the creation of 60 kV images from 130 kV input images^23^ and the creation of KV‐DRRs from MV‐DRs.^24^, ^25^ Although no identical benchmarks exist, our results fall within the range of reported metrics in related projectional image translation tasks, supporting the feasibility of our digital energy modulation framework as a viable approach for retrospective energy domain translation in projectional x‐ray imaging.

**TABLE 10:** Comparison of our energy modulation models' results and other projection x‐ray image translation study results.

Study/Methodology		Translation task	PSNR	SSIM	MSE	MAPE
*Energy modulation models based on DECT DRRs*	***CV w/ & woTB***	*polyhigh → 60 keV [80/20]*	*27.26 ± 0.43*	*0.920 ± 0.012*	*196.5 ± 19.2*	*9.9 ± 0.4*
		*polyhigh‐woTB → 60 keV‐woTB [80/20]*	*26.95 ± 0.34*	*0.916 ± 0.011*	*208.0 ± 26.7*	*10.2 ± 0.5*
		*polylow → 60 keV [80/20]*	*33.21 ± 0.38*	*0.961 ± 0.002*	*67.3 ± 7.1*	*5.3 ± 0.3*
		*polylow‐woTB → 60 keV‐woTB [80/20]*	*34.25 ± 0.38*	*0.971 ± 0.001*	*47.5 ± 4.7*	*4.6 ± 0.3*
	***CV DSR***	*polyhigh → polylow [80/20]*	*27.51 ± 0.54*	*0.923 ± 0.004*	*186.7 ± 23.9*	*9.3 ± 0.3*
		*polyhigh → polylow [40/20]*	*27.27 ± 0.40*	*0.917 ± 0.002*	*193.0 ± 25.4*	*9.4 ± 0.3*
		*polyhigh → polylow [20/20]*	*26.93 ± 0.41*	*0.910 ± 0.001*	*203.2 ± 25.1*	*10.1 ± 0.2*
	***CV PS***	*AP: polyhigh → polylow [11.4/2.85]*	*27.58 ± 0.47*	*0.911 ± 0.001*	*168.7 ± 25.9*	*8.5 ± 0.5*
		*LAT: polyhigh → polylow [11.4/2.85]*	*27.14 ± 0.51*	*0.908 ± 0.003*	*223.1 ± 31.9*	*12.3 ± 0.5*
Artificial intelligence‐based dual‐energy subtraction (AI‐DES)^23^		130 kV → 60 kV	33.80* ± *5.39	0.984* ± *0.006	–	–
		AI‐DES bone only images	21.10* ± *2.56	0.711* ± *0.055	–	–
		AI‐DES tissue only images	18.30* ± *1.97	0.433* ± *0.083	–	–
Dual energy x‐ray(DXR) bone suppression^20^		DXR (Radiograph) → bone suppression image	24.08	0.9304 (lung)	–	–
			28.582 (lung)			
Multi‐task organ segmentation and bone suppression in chest x‐ray radiography^21^		Rib suppression	–	^*^0.976* ± *0.006	^*^4.297* ± *1.0	–
				^*^MS‐SSIM	^*^RMSE	
Dual energy, like chest radiography image synthesis^19^		bone only images	23.89	0.771	^**^0.0647	–
		bone suppression images	33.77	0.918	^**^0.00042	–
Synthesizing kV‐DRRs from MV‐DRs^24,25^		MV‐DRs → KV‐DRRs	–	0.600	1395	–

Abbreviations: CV, cross‐validation; DSR, data size reduction; PS (AP/LAT), projection specific (Anterior‐Posterior / Lateral); and woTB, without TwinBeam data.

*Notes*: (1) The results of this study are italicized in the table at the top. The best‐performing model results for the three different experiments are bolded, underlined, and italicized. Train/Test split percentages are included in the translation task column at the end [train/test]. (2)*Values for the SSIM column for this study are for MS‐SSIM, and values for the MSE column are for RMSE.

(3)**Values for the MSE column for this study are for normalized MSE.

### 4.4 Future directions

Building on these insights, several avenues exist for advancing the digital energy modulation framework in projectional x‐ray imaging. Future work will investigate advanced deep learning architectures, such as diffusion and transformer models, that may better capture subtle attenuation differences and help address the underdetermined nature of projection imaging. Additionally, incorporating physics‐informed priors (e.g., tissue attenuation models or spectral characteristics) through regularization strategies could improve learning stability, generalizability, and interpretability.

Given the challenges of acquiring large‐scale, paired multi‐energy datasets in clinical settings, virtual imaging platforms offer a promising alternative. Monte Carlo simulation tools, anthropomorphic digital phantoms, and programmable x‐ray spectrum models could be used to create large, anatomically diverse datasets with controlled energy parameters and noise characteristics, enabling more robust training and evaluation pipelines.

While our proof of concept focused on the use of DECT‐derived DRRs, validating the framework on real‐world clinical radiographs, where energy parameters are known or controlled, will be essential. Radiologist assessment will play a key role in determining whether model‐generated images and ROIs support clinical decision‐making or streamline workflow. Rather than focusing solely on diagnostic outcomes, future evaluation should aim to quantify whether these tools improve interpretation efficiency, assist with secondary tasks such as image alignment in radiation therapy, or enhance clinician confidence in assessments.

Finally, as PCD technology becomes more widely adopted in CT and potentially in general radiography, it offers a scalable path to acquiring paired energy‐binned projection data without multiple exposures. This could provide ideal training datasets for future energy translation models, thereby expanding the clinical applications of digital energy modulation.

## 5 CONCLUSIONS

In summary, we explored the feasibility of using machine learning for digital energy modulation in projectional x‐ray imaging. By leveraging DECT‐derived DRRs, we trained and evaluated models capable of translating images between different energy domains. This framework provides a proof of concept for retrospective energy manipulation, which could allow for contrast enhancement without requiring multiple exposures.

Our results demonstrate that machine learning can be applied to energy translation in projectional x‐ray imaging, building upon prior work in projectional x‐ray image translation tasks. While previous studies have explored machine learning‐based transformations for bone/tissue decomposition in DER and kilovoltage‐megavoltage DRR translation, the proposed framework extends those concepts to retrospective energy domain translation for ROIs or whole images. Comparisons with previous studies in projectional x‐ray translation highlight the potential for this framework to generalize beyond DRRs to real‐world radiographic applications. The ability to modify subject contrast post‐acquisition across an entire image or within specific ROIs could offer clinicians a powerful tool for improving visualization in radiography, mammography, and fluoroscopy.

## AUTHOR CONTRIBUTIONS

*Study conception*: Richard Ryan Wargo and Siyong Kim. *Study design*: Richard Ryan Wargo, Siyong Kim, and William C. Sleeman IV. *Data analysis*: Richard Ryan Wargo. *Manuscript preparation*: Richard Ryan Wargo. *Critical review of manuscript*: Siyong Kim and William C. Sleeman IV.

## ETHICS STATEMENT

This study was granted exempt status by the institutional review board (IRB) for its retrospective analysis of imaging data.

## DECLARATION

During the preparation of the manuscript, the authors used ChatGPT to assist in proofreading. After using this tool, the authors reviewed and edited the content as needed and take full responsibility for the content of the publication.

## CONFLICT OF INTEREST STATEMENT

A patent application related to the concept and framework described in this manuscript has been filed by Virginia Commonwealth University, with Siyong Kim and Richard Ryan Wargo listed as inventors. The patent was not granted, and there are no licensing or commercial agreements in place.
